# Assessment of hepatic fibrosis in Egyptian children and adolescents with beta thalassemia major: a single center study

**DOI:** 10.1007/s00431-026-07170-4

**Published:** 2026-06-27

**Authors:** Marwa Abd Elhady, Mona Kaddah, Ahmed Marzouk, Engy Adel Mogahed, Noha Adel Yassin

**Affiliations:** 1 Department of Pediatrics, Cairo University, Cairo, Egypt; 2 Department of Tropical Medicine, Cairo University, Cairo, Egypt

**Keywords:** Beta thalassemia major, Fibrosis, Hepatic fibrosis

## Abstract

Liver fibrosis is a major complication in patients with transfusion-dependent beta-thalassemia, primarily driven by iron overload and suboptimal chelation therapy. This study aimed to assess the prevalence and severity of hepatic fibrosis in children with beta-thalassemia major using non-invasive modalities and to identify associated clinical and laboratory risk factors. This cross-sectional analytical study included 82 transfusion-dependent thalassemia patients. All participants underwent clinical evaluation, hematological and biochemical investigations, and liver fibrosis assessment using transient elastography (TE) (by FibroScan) and calculation of aspartate aminotransferase (AST)/platelet ratio index (APRI) and Fibrosis 4 score (FIB-4). Based on TE, 18 patients (22%) exhibited significant hepatic fibrosis (F2–F4). Both APRI and FIB-4 scores were significantly elevated in patients with significant fibrosis. Significant fibrosis was associated with older age (*p* = 0.019), longer disease duration (*p* = 0.015), heterogeneous liver echotexture on ultrasound (*p* < 0.001), poor adherence to chelation therapy (*p* < 0.001), and elevated transaminases and serum ferritin levels (*p* < 0.05). *Conclusion*: Significant hepatic fibrosis was observed in approximately one-fifth of the studied cohort of Egyptian children with beta-thalassemia major. It is strongly associated with older age, longer disease duration, iron overload, and inadequate chelation. TE, APRI, and FIB-4 are effective for early detection of hepatic fibrosis in these patients.

**Table:** 

**What is Known:** • *Hepatic fibrosis is a well-recognized complication of transfusion-dependent beta-thalassemia major, primarily resulting from chronic iron overload.* • *Liver biopsy remains the gold standard for fibrosis assessment; however, it is invasive. Non-invasive tools such as transient elastography, APRI, and FIB-4 have been increasingly used in adults, but limited data are available in pediatric populations, particularly in low- and middle-income countries.*	
**What is New:** • *This study shows that significant hepatic fibrosis affects approximately one in five Egyptian children with beta-thalassemia major.* • *It supports the clinical utility of FibroScan, APRI, and FIB-4 for fibrosis assessment in pediatric patients and highlights poor chelation adherence, iron overload, and longer disease duration as key modifiable risk factors.*

**What is Known:**

• *Hepatic fibrosis is a well-recognized complication of transfusion-dependent beta-thalassemia major, primarily resulting from chronic iron overload.*

• *Liver biopsy remains the gold standard for fibrosis assessment; however, it is invasive. Non-invasive tools such as transient elastography, APRI, and FIB-4 have been increasingly used in adults, but limited data are available in pediatric populations, particularly in low- and middle-income countries.*

**What is New:**

• *This study shows that significant hepatic fibrosis affects approximately one in five Egyptian children with beta-thalassemia major.*

• *It supports the clinical utility of FibroScan, APRI, and FIB-4 for fibrosis assessment in pediatric patients and highlights poor chelation adherence, iron overload, and longer disease duration as key modifiable risk factors.*

## Introduction

Beta-thalassemia is a hereditary blood disorder characterized by impaired synthesis of beta-globin chains of hemoglobin, resulting in a spectrum of clinical manifestations ranging from severe anemia to asymptomatic cases. Patients with β-thalassemia major typically present within the first 2 years of life and require regular red blood cell (RBC) transfusions to manage severe anemia [1].

In Egypt, approximately 1000 children are diagnosed with thalassemia annually among 1.5 million live births. The carrier rate in the population ranges from 5.3% to ≥ 9% [2]. Management of beta-thalassemia major relies primarily on lifelong RBC transfusions combined with iron chelation therapy. While transfusions are essential to correct anemia and suppress ineffective erythropoiesis, they lead to progressive iron accumulation, increasing the risk of complications such as cirrhosis, diabetes mellitus, cardiac disease, and hypogonadism [3].

Children with thalassemia are also at increased risk of acquiring hepatitis B and C infections through repeated transfusions. The severity of liver fibrosis is closely associated with iron overload [4], as well as with chronic viral hepatitis. In particular, chronic hepatitis C infection may progress to cirrhosis, hepatocellular carcinoma, and portal hypertension [5].

Liver biopsy remains the gold standard for staging hepatic fibrosis; however, it is invasive, costly, and associated with potential complications such as pain and bleeding, in addition to requiring hospitalization [6]. Transient elastography (using FibroScan®) has emerged as a reliable non-invasive alternative that measures liver stiffness by assessing the velocity of shear waves through hepatic tissue. It is a rapid, painless, and cost-effective method for evaluating liver fibrosis [4, 7].

Other non-invasive markers of liver fibrosis include the aspartate aminotransferase-to-platelet ratio index (APRI) and the Fibrosis 4 (FIB-4) score, both derived from routinely available laboratory parameters. APRI estimates fibrosis based on aspartate aminotransferase (AST) levels and platelet count, whereas FIB-4 incorporates AST, alanine aminotransferase (ALT), platelet count, and patient age. These indices are simple, cost-effective, and easily applicable in clinical settings, with acceptable diagnostic accuracy for fibrosis staging [8, 9].

Accordingly, this study aimed to evaluate the prevalence and severity of hepatic fibrosis in children with transfusion-dependent β-thalassemia major using non-invasive methods and to identify clinical and laboratory risk factors associated with its development.

## Methods

This cross-sectional study was conducted at the Pediatric Hematology Unit, Cairo University Children’s Hospital, Egypt, from February 2024 to August 2024. The study adhered to the principles of the Declaration of Helsinki. Written informed consent was obtained from the parents or legal guardians of all participants before enrollment. The study protocol was approved by the Institutional Review Board of Kasr Al-Ainy School of Medicine, Cairo University (Approval code: MS-545–2023).

The study included all children and adolescents below 18 years of age with established diagnosis of beta-thalassemia major who presented to the pediatric hematology unit during the time from February 2024 to August 2024. Diagnosis of beta-thalassemia major was based on the classic clinical picture and was confirmed by Hb electrophoresis or high-performance liquid chromatography. Patients were included only if they were clinically stable, afebrile, and lacked overt signs of acute infection. Transfusion dependency was defined as regular blood transfusions at intervals ranging from 15 to 45 days with a total number of transfusions exceeding 100 times. Children with any associated hematological disease were excluded from the study. Patients with cardiac diseases were excluded as well to eliminate hepatic congestion as a confounding factor of increasing hepatic stiffness in TE.

All participants underwent the following assessments:

- Medical history: demographic data, consanguinity, positive family history, disease duration, age at first blood transfusion, frequency of blood transfusions, time interval between blood transfusions, history of splenectomy, history of hepatitis C (HCV) or B virus (HBV) infection, and adherence to iron chelation therapy. Adherence to iron chelation was self-reported through direct interviews with patients and caregivers, who estimated the percentage of doses administered over the preceding 3 to 6 months. In the context of iron chelation therapy for thalassemia major, this refers specifically to the patient’s consistency in following the prescribed frequency, dosage, and schedule of chelating agents like deferoxamine, deferiprone, or deferasirox. Patients were classified as compliant or “adherent” if they take more than 75% of their prescribed doses. Non-compliance was defined as missing more than 25% of doses.
- Clinical examination: Comprehensive examination with emphasis on abdominal findings and anthropometric measurements.
- Laboratory investigations: Complete blood count (CBC), reticulocyte count, liver function tests, serum ferritin, HCV antibodies, and hepatitis B surface antigen (HBsAg), all performed at the time of enrollment.
- Non-invasive assessment tools for liver fibrosis: The FIB-4 = age (years) X AST IU/L/platelet count (10⁹/L) * ALT (IU/L)1/2. APRI = [{AST (IU/L)/Upper normal limit of AST (IU/L)}/Platelet count (10⁹/L)] *100 [10]. FIB-4 and APRI were calculated using the online calculator available at the University of Washington hepatitis C website: https://hepatitisc.uw.edu.
- Imaging: abdominal ultrasound and TE (FibroScan®, Echosens, Paris, France). Patients were fasted for at least 3 h prior to the test. TE examinations were performed by a single experienced operator. The liver stiffness was expressed in kilopascal (kPa). Fibrosis score was classified as follows: F0 to F1 (≤ 8.7 kPa) indicated either no or minimal liver scarring, F2 (8.8–9.5 kPa) indicated moderate scarring, F3 (9.6–14.4 kPa) indicated severe scarring, and F4 (≥ 14.5 kPa or higher) indicated liver cirrhosis [11]. Based on FibroScan results, patients were categorized into two groups: those without significant hepatic fibrosis (F0–F1) and those with significant hepatic fibrosis (F2–F4).

### Statistical methods

Statistical analysis was performed using SPSS software (version 21.0; IBM Corp., Armonk, NY, USA). Continuous variables (e.g., age and laboratory parameters) were presented as mean ± standard deviation (SD) for normally distributed data or median with interquartile range (IQR) for non-normally distributed data. Categorical variables (e.g., transfusion frequency, adherence to iron chelation therapy, and fibrosis stage) were expressed as frequencies and percentages.

Comparisons between groups were performed using Student’s *t*-test or Mann–Whitney *U* test for continuous variables, as appropriate, and the Pearson chi-square test or Fisher’s exact test for categorical variables. Correlations were assessed using Spearman’s rank correlation coefficient. A *p*-value < 0.05 was considered statistically significant.

## Results

The present study included 82 children with β-thalassemia major, of whom 49 were males (59.8%). The mean age was 13.35 ± 1.43 years (range, 12–18 years).

Based on transient elastography (FibroScan) findings, 18 patients (22%) had significant hepatic fibrosis (F2–F4), with liver stiffness values ranging from 8 to 41 kPa. Among them, seven patients (8.5%) had F2 fibrosis, six (7.3%) had F3 fibrosis, and five (6.1%) had F4 fibrosis.

Comparison between patients with significant fibrosis (F2–F4) and those without (F0–F1) revealed that patients with significant fibrosis were significantly older and had a longer disease duration, as well as shorter transfusion intervals. Poor adherence to iron chelation therapy was also significantly more frequent in this group. Other demographic and clinical characteristics showed no significant differences between the two groups (Table 1).

**Table 1:** Demographic data and clinical findings in children with beta-thalassemia major with and without significant hepatic fibrosis

Parameter	Patients without significant hepatic fibrosis (*n* = 64)	Patients with significant hepatic fibrosis (*n* = 18)	*p* value
Sex: number (%)			
Male	36 (56.3)	13 (72.2)	0.22
Female	28 (43.8)	5 (27.8)	
Age (years): mean ± SD	13.08 ± 1.09	14.33 ± 2	0.019^*^
Consanguinity: number (%)	51 (79.7)	17 (94.4)	0.29
Duration of illness (years): mean ± SD	12.23 ± 1.03	13.44 ± 1.85	0.015^*^
Age of first blood transfusion (months): mean ± SD	8.61 ± 3.26	9.33 ± 3.2	0.44
Time intervals between blood transfusions/month: mean ± SD	0.98 ± 0.08	0.92 ± 0.12	0.035*
Adherence to chelation therapy: number (%)	63 (98.4)	7 (38.9)	< 0.001^*^
Hepatitis C virus infection: number (%)	2 (3.1)	0	1.0
Hepatitis B virus infection: number (%)	0	0	
BMI (kg/m^2^): mean ± SD	15.84 ± 2.12	16.43 ± 1.57	0.27
Splenomegaly: number (%)	28 (43.8)	10 (55.6)	0.375
Splenectomy: number (%)	36 (56.3)	8 (44.4)	0.375

*BMI* body mass index, *SD* standard deviation

^*^p value is significant

**Fig. 1:**
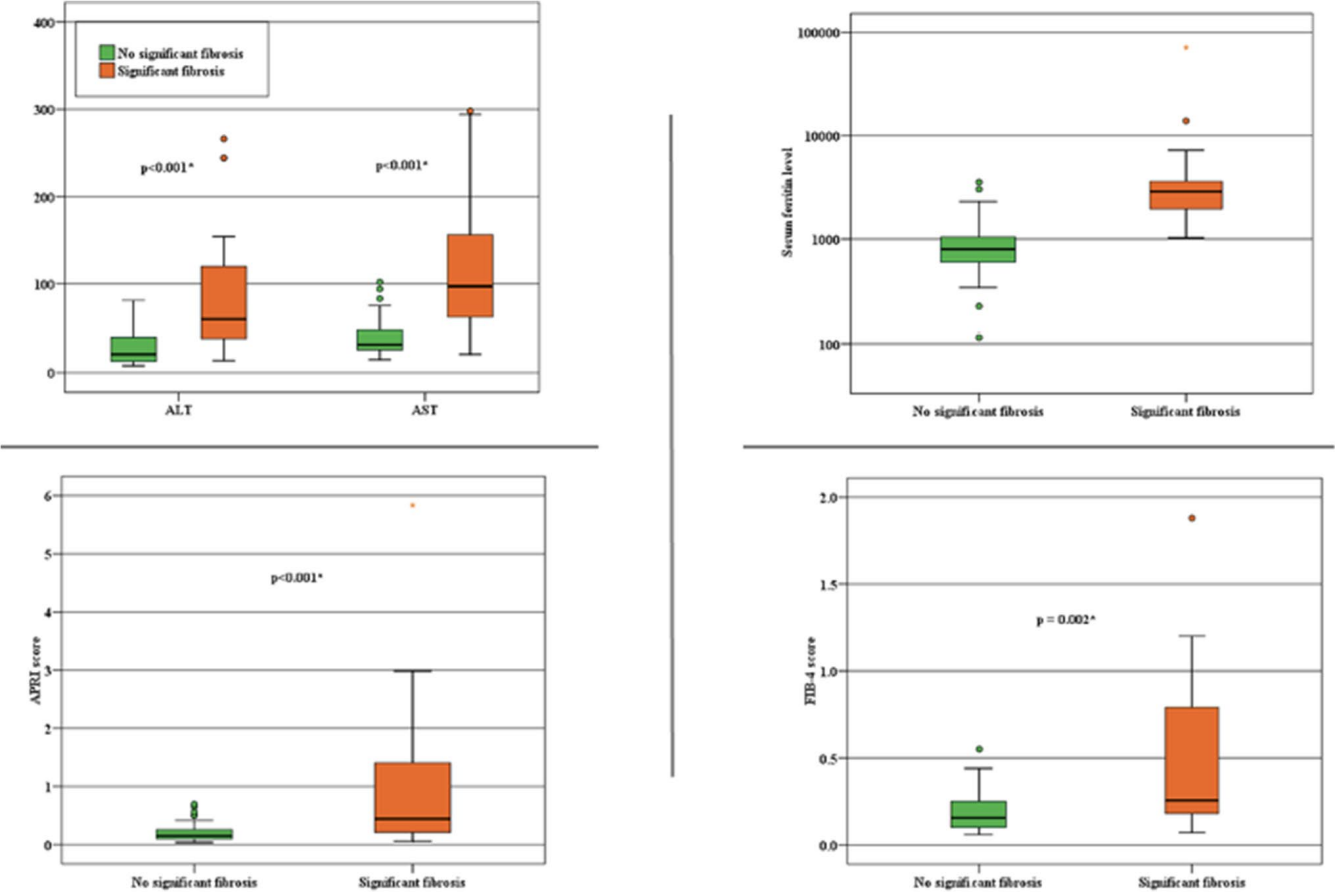
Comparison between thalassemic children with and without significant hepatic fibrosis regarding transaminases, ferritin level and fibrosis scores. The figure shows statistically increased median values of ALT, AST, ferritin, APRI, and FIB-4 scores in thalassemic children with significant hepatic fibrosis. ALT, alanine aminotransferase; APRI, aspartate aminotransferase to platelet ration index; AST, aspartate aminotransferase; FIB-4, fibrosis 4

Regarding laboratory findings, patients with significant hepatic fibrosis exhibited significantly higher total leukocyte counts, liver enzyme levels, serum ferritin, APRI, and FIB-4 scores. In contrast, hemoglobin and bilirubin levels did not differ significantly between the groups. Additionally, a heterogeneous liver echotexture on abdominal ultrasound was significantly more common among patients with significant fibrosis (Table 2, Fig. 1).

**Table 2:** Laboratory parameters and fibrosis scores in children with beta-thalassemia major with and without significant hepatic fibrosis

Parameter	Patients without significant hepatic fibrosis (*n* = 64)	Patients with significant hepatic fibrosis (*n* = 18)	*p* value
Hemoglobin (g/dl): mean ± SD	7.26 ± 0.96	7.26 ± 0.64	0.989
TLC (× 10^3^/l): median (IQR)	12 (7–19.8)	16.95 (12.8–23)	0.049^*^
PLT (× 10^3^/l): median (IQR)	522 (352.5–861)	627 (258–940)	0.911
Reticulocyte count: mean ± SD	2.08 ± 0.95	2.81 ± 1.77	0.145
Total bilirubin (mg/dl): median (IQR)	1.4 (1.2–2.2)	1.95 (1.3–2.4)	0.189
Direct bilirubin (mg/dl): median (IQR)	0.3 (0.3–0.5)	0.4 (0.3–0.5)	0.128
ALT (IU/L): median (IQR)	21 (13–40.5)	59.5 (39–120)	< 0.001^*^
AST (IU/L): median (IQR)	32 (26–47)	97 (62–156)	< 0.001^*^
ALP (U/L): mean ± SD	179.5 ± 64.72	223 ± 50.55	0.012^*^
GGT (IU/L): mean ± SD	13.5 ± 4.64	48.89 ± 30.74	< 0.001^*^
Albumin (g/dl): mean ± SD	3.13 ± 0.33	3.14 ± 0.28	0.958
INR: mean ± SD	1.15 ± 0.17	1.27 ± 0.12	0.001^*^
Serum ferritin (ng/ml)			
- Median (IQR)	802 (599.5–1048.5)	2506.5 (1940–3451)	< 0.001^*^
- Range	115–3562	1032–7500	
Number of patients with serum ferritin > 1500 ng/ml (%)	6 (9.4)	15 (83.3)	0.001^*^
APRI: median (IQR)	0.152 (0.98–0.258)	0.443 (0.211–1.405)	< 0.001^*^
FIB-4 score: median (IQR)	0.155 (0.1–0.25)	0.255 (0.18–0.79)	0.002^*^
Number of patients with heterogenous liver in ultrasound (%)	3 (4.7)	13 (72.2)	< 0.001^*^

*ALT* alanine aminotransferase, *ALP* alkaline phosphatase, *APRI* aspartate aminotransferase to platelet ration index, *AST* aspartate aminotransferase, *FIB-4* fibrosis 4, *GGT* gamma glutamyl transpeptidase, *INR* international normalized ratio, *IQR* interquartile range, *PLT* platelets, *SD* standard deviation, *TLC* total leukocyte count

^*^p value is significant

Correlation analysis demonstrated a significant positive correlation between liver stiffness (kPa) and patient age, disease duration, transaminase levels, reticulocyte count, serum ferritin, and fibrosis indices (APRI and FIB-4). Conversely, the interval between blood transfusions was negatively correlated with liver stiffness (Table 3).

**Table 3:** Correlation between degree of liver fibrosis measured by FibroScan and other parameters

	Degree of hepatic fibrosis (kPa)	
	Spearman coefficient (***r***)	*p* value
Age (years)	0.357	0.001^*^
Disease duration (years)	0.362	0.001^*^
Age at first blood transfusion (months)	0.211	0.057
Time intervals between blood transfusions (days)	− 0.381	< 0.001^*^
Hemoglobin (g/dl)	− 0.121	0.278
TLC (× 10^3^/l)	0.089	0.427
PLT (× 10^3^/l)	0.046	0.684
Reticulocyte count	0.302	0.006^*^
Total bilirubin (mg/dl)	0.034	0.760
Direct bilirubin (mg/dl)	0.098	0.379
ALT (IU/L)	0.501	< 0.001^*^
AST(IU/L)	0.551	< 0.001^*^
ALP (U/L)	0.176	0.113
GGT (IU/L)	0.467	< 0.001^*^
Albumin (g/dl)	− 0.057	0.612
INR	0.622	< 0.001^*^
Serum ferritin (ng/ml)	0.622	< 0.001^*^
APRI	0.262	0.017^*^
FIB-4 score	0.355	0.001^*^

*ALT* alanine aminotransferase, *ALP* alkaline phosphatase, *APRI* aspartate aminotransferase to platelet ration index, *AST* aspartate aminotransferase, *FIB-4* fibrosis 4, *GGT* gamma glutamyl transpeptidase, *INR* international normalized ratio, *PLT* platelets, *TLC* total leukocyte count

^*^p value is significant

Receiver operating characteristic (ROC) curve analysis showed that serum ferritin had the highest diagnostic performance for predicting significant fibrosis, with an area under the curve (AUC) of 0.947 (95% CI 0.902–0.993; *p* < 0.001). At a cutoff value > 1173 ng/mL, serum ferritin demonstrated a sensitivity of 94.44% and a specificity of 82.81%, with a high negative predictive value (98.1%).

The univariate analysis showed significant correlation between hepatic fibrosis and other parameters including age, duration of illness, frequency of blood transfusion, compliance to chelation therapy, ALT, AST, serum ferritin level, FIB-4 score, and APRI. However, in the multivariate analysis, only compliance to chelation therapy (OR = 35.334, *p* = 0.042) and serum ferritin level (OR = 1.002, *p* = 0.015) remained significant predictors of fibrosis (Table 4).

**Table 4:** Univariate and multivariate logistic regression analysis for the parameters affecting hepatic fibrosis in children with beta-thalassemia major

	Univariate analysis		^#^Multivariate analysis	
	*p*	OR (LL–UL 95% C.I)	*p*	OR (LL–UL 95% C.I)
Age (years)	0.003^*^	1.802 (1.225–2.652)	0.326	3.128 (0.321–30.482)
Duration of disease (years)	0.003^*^	1.918 (1.257–2.926)	0.973	1.046 (0.079–13.818)
Frequency of blood transfusion (months)	0.015^*^	0.001 (0.0–0.275)	0.698	0.076 (0.0–33251.9)
Compliance to chelation therapy	< 0.001^*^	99 (11.069–885.462)	0.042^*^	35.334 (1.131–1103.7)
ALT	0.001^*^	1.043 (1.018–1.069)	0.113	0.886 (0.762–1.029)
AST	< 0.001^*^	1.055 (1.027–1.083)	0.079	1.113 (0.988–1.254)
Serum ferritin level	< 0.001^*^	1.002 (1.001–1.003)	0.015^*^	1.002 (1.000–1.004)
FIB-4 score	0.007^*^	121.831 (3.829–3875.92)	0.252	0.177 (0.009–3.423)
APRI	0.002^*^	80.239 (4.733–1360.224)	0.433	2.365 (0.276–20.282)

*APRI* aspartate aminotransferase to platelet ration index, *AUC* area under the curve, *CI* confidence interval, *FIB-4* fibrosis 4, *LL* lower limit, *OR* odd’s ratio, *UL* upper limit

^*^p value is significant

^#^All variables with *p* < 0.05 were included in the multivariate analysis

## Discussion

Beta-thalassemia major is a severe inherited hemoglobinopathy that requires lifelong transfusion therapy, predisposing patients to iron overload and subsequent organ complications, particularly hepatic fibrosis [12]. The burden of liver disease in thalassemic children is further amplified in regions such as Egypt, where high disease prevalence and limited access to early screening contribute to underdiagnosis of hepatic complications [13].

Traditionally, liver biopsy has been considered the gold standard for staging hepatic fibrosis; however, its invasive nature, cost, and associated risks have necessitated the use of reliable non-invasive alternatives [14]. In this context, the present study evaluated hepatic fibrosis among Egyptian children with beta-thalassemia major using TE (FibroScan®) alongside biochemical markers including APRI and FIB-4 scores. In low–middle income countries such as Egypt, TE is considered a cost-effective method for assessing fibrosis. It is available as a free or minimal charge service in the main governmental Egyptian hospitals. This can help tailor our personalized medical service. Our findings demonstrated that 22% of the cohort had significant fibrosis (F2–F4), with elevated serum ferritin levels and poor adherence to iron chelation therapy emerging as key predictors. These results are consistent with previously reported prevalence rates of hepatic fibrosis in thalassemic populations, supporting the diagnostic utility of transient elastography in this setting [15].

In the current study, patients with significant fibrosis were older, had longer disease duration, shorter transfusion intervals, and lower adherence to iron chelation therapy. Similar findings have been reported in previous studies, where fibrosis severity was significantly associated with increasing age, reflecting cumulative iron overload over time [16–18]. These findings underscore the importance of strict adherence to iron chelation therapy, which should be reinforced through continuous patient and caregiver education.

Liver enzyme levels were significantly elevated in the fibrosis group (*p* < 0.001), indicating ongoing hepatic injury and inflammation. This observation aligns with prior studies demonstrating a positive association between elevated transaminases and the degree of hepatic fibrosis in children with β-thalassemia major [19].

In our study, serum ferritin showed the highest diagnostic performance for detecting significant fibrosis, with an AUC of 0.947 at a cutoff value > 1173 ng/mL, yielding a sensitivity of 94.44% and specificity of 82.81%. These results indicate excellent discriminative ability and are consistent with previous studies reporting serum ferritin as a strong surrogate marker of iron overload and hepatic fibrosis progression in transfusion-dependent patients [4].

It is well-established that inflammation or occult infection can elevate ferritin levels independently of iron stores. While C-reactive protein was not measured in this cohort, we attempted to mitigate this confounding factor by only enrolling patients who were clinically stable, afebrile, and lacked overt signs of acute infection at the time of blood sampling. Ferritin utility should be interpreted with caution in the presence of inflammatory states. The lack of inflammatory markers assessment is considered one of our study limitations.

APRI and FIB-4 were significantly higher in the significant fibrosis group. This agrees with other studies that validated APRI and FIB-4 as a feasible alternative to biopsy in detecting fibrosis in iron overload-associated liver diseases and a good non-invasive indicator of progressive liver fibrosis in children [20–22].

Regarding clinical factors, splenomegaly and a history of splenectomy were not significantly associated with hepatic fibrosis severity in our study. This finding contradicts earlier assumptions that splenectomy may influence hepatic outcomes by altering hematologic parameters and iron metabolism. However, the impact of splenic status on liver disease progression in beta-thalassemia remains controversial and may depend more on the timing and clinical indication for splenectomy rather than splenic status alone [23, 24].

In the present cohort, no cases of HBV infection were identified, whereas HCV infection was detected in only two patients, none of whom exhibited significant fibrosis. The very low prevalence of viral hepatitis in this cohort minimizes its potential confounding effect on liver pathology, supporting the conclusion that hepatic fibrosis in this population is primarily driven by iron overload rather than coexisting viral infection.

Despite the important findings of this study, several limitations should be acknowledged. First, the relatively small sample size may limit the generalizability of the results. Second, liver biopsy—the gold standard for assessing hepatic fibrosis—was not performed due to ethical and clinical considerations in this pediatric population; therefore, non-invasive methods (TE and serum-based indices) were used as the primary diagnostic tools.

In addition, some variables included in fibrosis scoring systems, such as platelet count and AST levels, may be influenced by factors unrelated to fibrosis, including hypersplenism or a recent blood transfusion, which could affect the accuracy of APRI and FIB-4. Furthermore, although serum ferritin was a strong predictor of fibrosis in this study, it can be affected by acute or chronic inflammatory states, which were not systematically excluded. Finally, the cross-sectional design limits assessment of fibrosis progression over time, highlighting the need for longitudinal studies to evaluate disease evolution and long-term outcomes.

Lastly, as this is a single-center study, the findings cannot be generalized to the broader thalassemia population in Egypt and need external validity by larger multi-center studies.

In conclusion, significant hepatic fibrosis was observed in about one fifth of the studied cohort of children with beta-thalassemia major. Fibrosis was strongly associated with older age, longer disease duration, elevated liver enzymes, increased serum ferritin levels, and poor adherence to iron chelation therapy.

Non-invasive tools for assessing hepatic fibrosis in thalassemic children such as TE, APRI, and FIB-4 demonstrated valuable utility for screening, early detection, and risk stratification particularly in resource-limited settings. Multidisciplinary management involving hematologists, hepatologists, and nutrition specialists is essential to optimize care and reduce hepatic complications in this high-risk population.

## Data Availability

No datasets were generated or analysed during the current study.

## Abbreviations

ALP: Alkaline phosphatase
ALT: Alanine aminotransferase
APRI: Aspartate Aminotransferase to Platelet Ratio Index
AST: Aspartate aminotransferase
AUC: Area under the curve
BMI: Body mass index
CBC: Complete blood count
FIB-4: Fibrosis-4
GGT: Gamma glutamyl transpeptidase
HBV: Hepatitis B virus
HCV: Hepatitis C virus
INR: International normalized ratio
IQR: Interquartile range
kPa: Kilopascal
LL: Lower limit
OR: Odds ratio
PLT: Platelets
RBC: Red blood cell
SD: Standard deviation
TE: Transient elastography
TLC: Total leucocyte count
UL: Upper limit
